# Vaccine Interaction and Protection against Virulent Avian Metapneumovirus (aMPV) Challenge after Combined Administration of Newcastle Disease and aMPV Live Vaccines to Day-Old Turkeys

**DOI:** 10.3390/vaccines11030708

**Published:** 2023-03-22

**Authors:** Caterina Lupini, Matteo Legnardi, Giulia Graziosi, Mattia Cecchinato, Valeria Listorti, Calogero Terregino, Elena Catelli

**Affiliations:** 1 Department of Veterinary Medical Sciences, University of Bologna, 40064 Ozzano dell’Emilia, Italy; 2 Department of Animal Medicine, Production and Health, University of Padova, 35020 Legnaro, Italy; 3 Struttura Semplice Section of Genoa-Portualità, Istituto Zooprofilattico Sperimentale del Piemonte, Liguria e Valle d’Aosta, 16129 Genoa, Italy; 4 OIE/FAO and National Reference Laboratory for Newcastle Disease and Avian Influenza, Istituto Zooprofilattico Sperimentale delle Venezie, 35020 Legnaro, Italy

**Keywords:** avian metapneumovirus, Newcastle disease virus, vaccine interaction, Turkeys

## Abstract

Newcastle disease virus (NDV) and avian metapneumovirus (aMPV) are among the most impactful pathogens affecting the turkey industry. Since turkeys are routinely immunized against both diseases, the hatchery administration of the combined respective live vaccines would offer remarkable practical advantages. However, the compatibility of NDV and aMPV vaccines has not yet been experimentally demonstrated in this species. To address this issue, an aMPV subtype B live vaccine was administered to day-old poults either alone or in combination with one of two different ND vaccines. The birds were then challenged with a virulent aMPV subtype B strain, clinical signs were recorded and aMPV and NDV vaccine replication and humoral immune response were assessed. All results supported the absence of any interference hampering protection against aMPV, with no significant differences in terms of clinical scoring. In addition, the mean aMPV vaccine viral titers and antibody titers measured in the dual vaccinated groups were comparable or even higher than in the group vaccinated solely against aMPV. Lastly, based on the NDV viral and antibody titers, the combined aMPV and NDV vaccination does not seem to interfere with protection against NDV, although further studies involving an actual ND challenge will be necessary to fully demonstrate this hypothesis.

## 1. Introduction

The turkey industry is a major contributor to worldwide meat production, having yielded approximately 6 million tons of meat in 2020 alone [1]. However, the efficiency and sustainability of the sector are threatened by infectious diseases, making vaccination programs and biosecurity essential to limit the occurrence and impact of outbreaks. Among the most relevant viral diseases against which turkeys are routinely immunized are Newcastle disease (ND) and turkey rhinotracheitis (TRT).

ND is caused by virulent strains of avian paramyxovirus 1 (APMV-1), which belongs to the family *Paramyxoviridae*, genus *Orthoavulavirus*. The disease manifestation may include a range of respiratory, nervous, enteric, and reproductive signs depending on the strain involved along with factors related to the host. Turkeys are susceptible to all NDV pathotypes, albeit the disease is generally less severe than in chickens [2]. In addition to the direct damages due to its high mortality and morbidity, the devastating economic losses caused by ND are also related to the costs of eradication programs and trade restrictions applied in countries concerned about outbreaks [3].

TRT is a disease characterized by aspecific respiratory and reproductive problems whose severity and related mortality are largely influenced by secondary bacterial infections [4]. TRT is caused by avian metapneumovirus (aMPV), an RNA virus belonging to the family *Pneumoviridae*, genus *Metapneumovirus*. Turkeys represent the main host of aMPV, as they are susceptible to all four historically recognized aMPV subtypes, named A, B, C (further divided into two lineages, one adapted to turkeys and the other to ducks), and D [5]. Nowadays, there is consensus about the predominance of subtype C in North America and subtype B in the rest of the world [4,6]. Nonetheless, two novel subtypes have recently been identified in gulls [7] and parakeets [8] for which the susceptibility of turkeys is unknown.

Both NDV and aMPV are kept under control in turkey farms by administering live vaccines during the first days of life, usually observing an interval between the two vaccinations to avoid any possible interference. A vaccination program allowing the simultaneous administration of both vaccines in the hatchery would have several practical, economic, and biosecurity advantages, but such a change would require an assessment of the compatibility between the vaccines against the two pathogens. Although the absence of significant interference is supported by several studies [9,10,11,12,13], all these experiments have been conducted in chickens and not in turkeys [14]. The two species show different susceptibility and immune responses against the two diseases [3,5], requiring a separate evaluation. The aim of the present study, therefore, is to evaluate, under experimental conditions, the interaction between an aMPV vaccine and two different NDV vaccine strains when coadministered to day-old turkeys, assessing potential changes in terms of coverage, immune response, and achieved clinical protection following aMPV challenge.

## 2. Materials and Methods

### 2.1. Birds

Eighty unvaccinated day-old poults were obtained from an Italian commercial turkey hatchery adhering to high standards of biosecurity.

### 2.2. Vaccines

The following commercially available live vaccines were administered, alone or in combination: Rinovax^®^ (Boehringer Ingelheim Animal Health, Ingelheim am Rhein, Germany), based on aMPV subtype B vaccine strain VCO3; Bio B1^®^ (Boehringer Ingelheim Animal Health, Ingelheim am Rhein, Germany), based on NDV vaccine strain B1; and Avinew^®^ (Boehringer Ingelheim Animal Health, Ingelheim am Rhein, Germany), based on NDV vaccine strain VG/GA. The vaccines were reconstituted according to the manufacturer’s guidelines and administered by the ocular route. The dosages used were 10^2.3^ TCID_50_ for Rinovax^®^, 10^6^ EID_50_ for Bio B1^®^, and 10^5.5^ EID_50_ for Avinew^®^.

### 2.3. Challenge Virus

Virulent aMPV subtype B strain IT/Ty/Vr240/87, used as the challenge virus, titrated in tracheal organ culture [15], and the 50% endpoint was calculated with the Reed-Muench method [16]. The challenge dose, administered by the ocular route, was 3.77 log_10_ CD_50_.

### 2.4. Experimental Trial

The trial was performed in HM 1500 biological isolation units (Montair Process Technology BV, Kronenberg, The Netherlands) in the facilities of the Department of Veterinary Medical Sciences of the University of Bologna. The turkeys were individually numbered, divided into six groups, and vaccinated at one day of age as follows:

- TRT group (16 birds)—vaccinated with aMPV strain VCO3;
- B1 group (8 birds)—vaccinated with NDV strain B1;
- VG/GA group (8 birds)—vaccinated with NDV strain VG/GA;
- TRT-B1 group (16 birds)—vaccinated with aMPV strain VCO3 and NDV strain B1, coadministered;
- TRT-VG/GA group (16 birds)—vaccinated with aMPV strain VCO3 and NDV strain VG/GA, coadministered;
- CONTROL group (16 birds)—mock-vaccinated with sterile water.

The trial lasted 32 days, providing water and feed ad libitum. Eight oro-pharyngeal swabs per group were collected at 2, 4, 6, 8, 10, 14, 18, 22, 26, and 30 days post-vaccination (p.v.) to investigate the colonization and replication of vaccine viruses in target tissues by real-time reverse transcription-PCR (qRT-PCR). Eight blood samples per group were collected from the cutaneous ulnar vein at 7, 14, 21, and 28 days p.v. for the detection of anti-aMPV antibodies by ELISA and anti-NDV antibodies by hemagglutination inhibition (HI). In groups composed of 16 birds, the birds to be sampled were selected randomly at the first sampling point and then followed longitudinally.

On day 21 p.v., eight randomly chosen animals from groups TRT, TRT-B1, TRT-VG/GA, and CONTROL were moved to separate isolation units and challenged by administering the challenge virus by eye drop. From day 1 post-infection (p.i.) until the end of the trial, the birds were monitored daily for clinical signs, assigning a standardized score to each bird.

### 2.5. aMPV and NDV Detection in Oro-Pharyngeal Swabs

RNA was extracted from individual dry swabs using the guanidinium isothiocyanate method, as described by Li et al. [17]. aMPV was amplified using a quantitative real-time RT-PCR assay involving the use of primers designed on the gene coding for the small hydrophobic (SH) protein and a molecular beacon probe specific for aMPV subtype B, designed by Cecchinato et al. [18]. aMPV titers, expressed as cyliostatic doses (CD_50_)/mL, were calculated by absolute quantification. NDV detection was carried out with the real-time RT-PCR method designed by Wise et al. [19]. The sequences of the primers and probes are detailed in Table 1.

### 2.6. Serology for aMPV and NDV

Anti-aMPV antibody titers were assessed using the commercial ELISA kit Flockchek^®^ APV Ab, (IDEXX Laboratories, Milano, Italy), following the manufacturer’s instructions. Anti-NDV antibody titers were determined by the HI test following the method described by the World Organization for Animal Health [20].

### 2.7. Clinical Signs

Clinical signs were scored following the scale proposed by Naylor and Jones [21], as outlined below:

- 0: no signs;
- 1: clear nasal exudate;
- 2: turbid nasal exudate;
- 3: swollen infra-orbital sinuses and/or frothy eyes.

Minimal nasal pressure was applied to facilitate the extrusion of exudates. A score of 2 or above was considered a reliable marker of disease which would usually lead to the development of severe signs in field conditions.

### 2.8. Statistical Analysis

The existence of significant differences in the number of NDV and aMPV-positive samples among different groups was investigated using Fisher’s exact test (SPSS, IBM). Differences in terms of aMPV viral titers and antibody titers between groups were investigated at each time point using the Kruskal–Wallis test followed by the Wilcoxon rank sum test as a post-hoc test (SPSS, IBM). The comparison between groups based on cumulative clinical scores measured after the aMPV challenge was performed using the Wilcoxon rank sum test (SPSS, IBM). For all tests, the significance level was set to *p* < 0.05.

## 3. Results

### 3.1. Shedding of Vaccine Viruses

The number of NDV-positive birds per day of sampling was comparable in all vaccinated groups. All the investigated birds tested positive at least once by four days p.v. and the number of positive birds/groups decreased sharply after 10 days p.v. (Table 2). The total number of birds positive with the qRT-PCR for aMPV differed slightly among the vaccinated groups. All the investigated birds tested positive at least once by 14 days p.v. No statistically significant differences were observed in the count of NDV and aMPV-positive samples among different groups.

**Figure 1:**
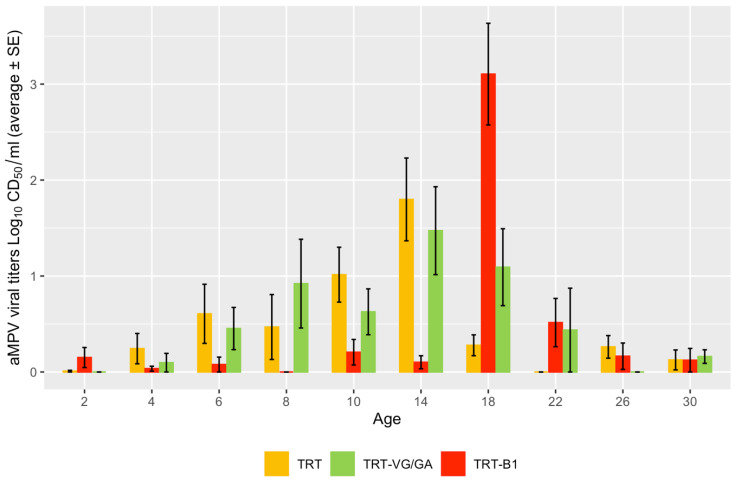
Mean aMPV vaccine load assessed longitudinally in oro-pharyngeal swabs using quantitative real-time RT-PCR.

The mean aMPV vaccine load measured in oro-pharyngeal swabs at each time point is reported in Figure 1. Statistically significant differences were found only at day 18 (*p* = 0.0021) and were attributable to the higher titers measured in the TRT-B1 group compared to both the TRT (*p* = 0.0026) and TRT-VG/GA (*p* = 0.0069) groups.

### 3.2. Serology

**Figure 2:**
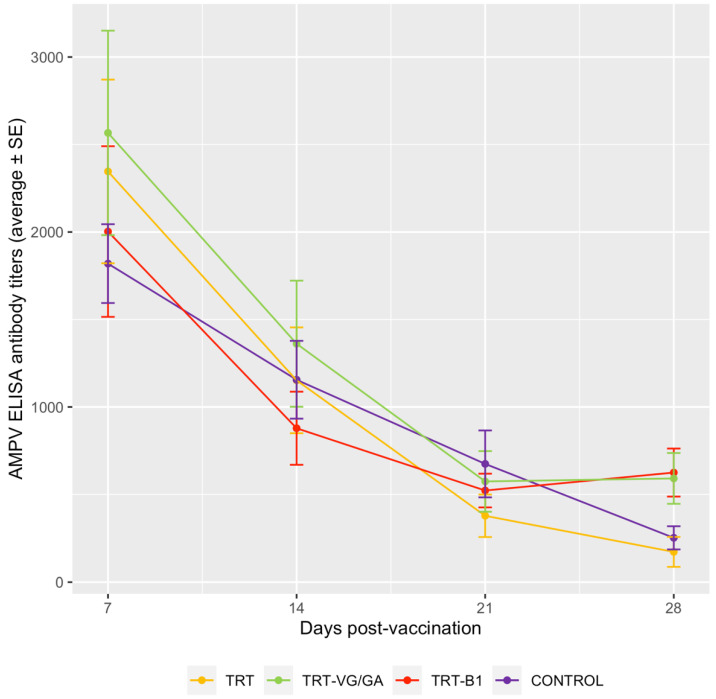
Mean anti-aMPV antibody titers measured by ELISA in aMPV-vaccinated turkeys and the control group.

No statistically significant differences in mean anti-aMPV antibody titers were found at 7, 14, and 21 days p.v., but only at 28 days p.v. (*p* = 0.0121), when groups vaccinated with aMPV and NDV vaccines showed higher titers than both the CONTROL group (CONTROL vs. TRT-B1: *p* = 0.0411; vs. TRT-VG/GA: *p* = 0.0205) and the group which received the aMPV vaccine alone (TRT vs. TRT-B1: *p* = 0.0424; vs. TRT-VG/GA: *p* = 0.0262) (Figure 2).

**Figure 3:**
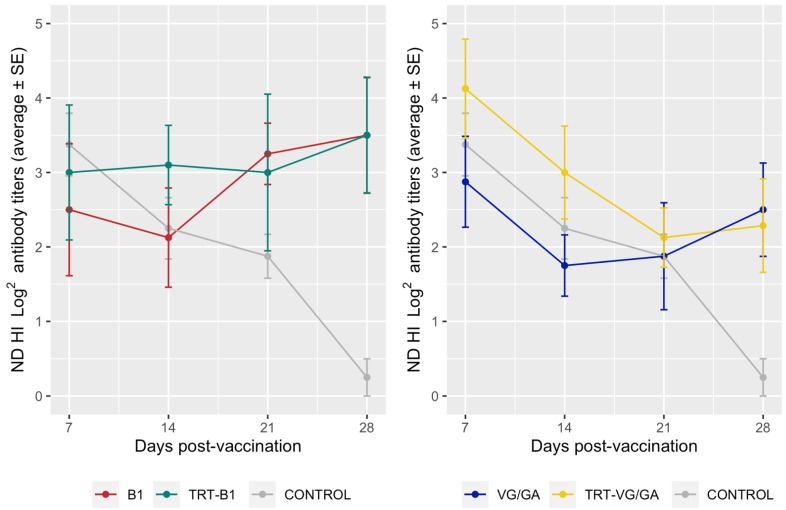
NDV-HI titers measured in groups vaccinated with vaccine strains B1 (**left**) and VG/GA (**right**) compared to the control group.

The mean NDV-HI titers also did not show significant differences between groups at any time points except for day 28 p.v. (*p* = 0.0224), when the CONTROL group differed from all vaccinated groups (CONTROL vs. B1: *p* = 0.04435; vs. TRT-B1: *p* = 0.0043; vs. VG/GA: *p* = 0.0105; vs. TRT-VG/GA: *p* = 0.0184) (Figure 3). No statistically significant differences were observed between groups that received NDV vaccines alone and those which were also vaccinated against aMPV.

### 3.3. Clinical Signs after aMPV Challenge

**Figure 4:**
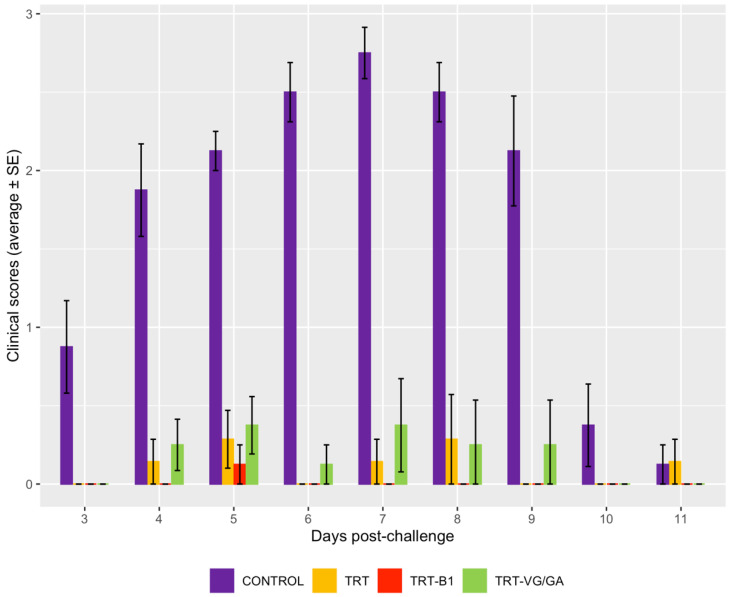
Mean clinical scores observed longitudinally in groups challenged with a virulent aMPV strain IT/Ty/Vr240/87.

After challenge with a field aMPV strain, the unvaccinated birds in the CONTROL group showed clinical signs with a total score of 15.25, significantly higher than all vaccinated groups (*p* < 0.001 in all cases). No statistically significant differences in clinical scores were found between the TRT, TRT-VG/GA, and TRT-B1 groups (Figure 4).

## 4. Discussion

The present study constitutes the first assessment of the potential implications of coadministering aMPV and NDV vaccines in turkeys. Both the aMPV vaccine strain and the challenge virus belonged to subtype B, a choice motivated by its relevance in the global epidemiological landscape. Historically, the two subtypes with the greatest diffusion are A and B, commonly found in Europe, Asia, Africa, and South America, whereas subtype C strains affecting turkeys are only reported in North America and subtype D has only ever been detected in archive samples [4]. In recent years, however, detections of subtype A diminished significantly, at least in Europe, and subtype B consolidated its status as the predominant field threat [6]. It is also worth noting that most of the commercially available vaccines are based on subtype B and that heterologous cross-protection has been established for both chickens and turkeys [22,23], suggesting that the conclusions reached in the present study could be considered relevant in most of the epidemiological scenarios encountered around the world.

No relevant differences were noted in terms of vaccine coverage, as the highest number of positive birds was reached between 10 and 18 days p.v. both in the single and the combined vaccination groups. When looking at aMPV viral titers, however, the coadministration with NDV vaccines appears to have resulted in a more intense, albeit slightly delayed, replication of the aMPV vaccine strain in the respiratory tract. Some differences were observed based on the NDV vaccine used, with the B1-based vaccine leading to a higher peak (18 days p.v.) in aMPV titers compared to the VG-GA one. Such an improvement in aMPV replication in dually vaccinated groups is not immediate to justify. In previous studies, the coadministration with vaccines against another notable respiratory virus, infectious bronchitis virus (IBV), was found to lower aMPV vaccine replication [24], whereas the combined day-old vaccination against aMPV and NDV seemed to only delay it, possibly because NDV proliferates more quickly and hampers aMPV replication in the first days [9,10]. Even though aMPV presence was only assessed by RT-PCR and not quantified, preventing a direct comparison, the detection of aMPV up to 24 days after dual vaccination [9,10] appears consistent with the results of the present study.

No differences in aMPV serological response could be observed between all groups up to 21 days p.v. The titers observed until then, which show a steadily declining trend, are due to the persistence of maternally derived antibodies (MDAs). In commercial turkeys such as the ones used in this study, such levels of MDAs are expected and, albeit insufficient to protect against the disease, are not considered detrimental for aMPV vaccination [25]; in fact, their presence is a key factor to replicate a typical field situation in experimental conditions. Starting from 28 days p.v., while aMPV antibodies kept decreasing in the single vaccinated and the control group, the two combinedly vaccinated groups showed higher and comparable ELISA titers. This may be seen as a direct consequence of the more intense aMPV vaccine replication observed in these two groups, which may have elicited a greater antibody response.

Previous studies conducted in chickens reported variable effects of dual vaccination on aMPV humoral response. In SPF birds, anti-aMPV antibody titers were diminished by the coadministration of vaccines against aMPV and NDV [9] or aMPV, NDV, and IBV [12,13]. Ganapathy et al. [11] reported comparable antibody titers in single and dual-vaccinated birds, while higher titers were observed in aMPV and NDV-vaccinated chickens with MDAs against NDV [10]. Despite these differences, however, all the mentioned studies are in agreement regarding the clinical protection against TRT, which was not impacted by the variability in aMPV immune response. This is likely because, besides humoral immunity, the protection against aMPV also relies on local and cell-mediated mechanisms [26]. In the present study, the coadministration of aMPV and NDV vaccines was confirmed not to alter the protection against an aMPV challenge, with the clinical scores of all vaccinated groups being similar and significantly lower than the control group.

Although the protection against an NDV challenge was not assessed directly, a comparable NDV humoral response was found in groups coadministered with NDV and aMPV vaccines and in those immunized only against NDV. Similar results were found for both NDV strain B1, that exhibits a preferential tropism for the respiratory tract, and strain VG/GA, whose tropism is mainly enteric [27]. Previous studies reported that coadministering aMPV and NDV vaccines resulted in a temporary boost in NDV antibodies in SPF chickens [9] but not in broilers having anti-NDV MDAs, which likely contained the initial NDV vaccine replication [10] and were clearly present in the poults tested in this study. The absence of differences between the single and dually vaccinated groups in terms of antibody response, which is recognized to play a key role in ND protection [28], allows for supporting the efficacy of NDV vaccination even when administered along with aMPV vaccines.

Considering the threat posed by ND and TRT to the turkey sector and the wide adoption of the respective vaccines, the relevance of the present study appears evident. Based on the obtained findings, the concurrent administration of an aMPV vaccine based on strain VCO3 and B1 or VG/GA-based NDV vaccines appears fully applicable, with no interference with the conferred clinical protection against aMPV. In addition, dual vaccination protocols even led to an improved aMPV vaccine replication and immunological response, while ND vaccine coverage and elicited antibody titers were unaffected. These results open up the possibility to apply both aMPV and NDV vaccines to day-old poults, bringing clear practical benefits to the daily routine. In fact, reducing the number of administrations would allow for cutting costs, reduce the stress placed on birds due to handling procedures, and improve biosecurity, ultimately enabling a more effective and convenient strategy against two burdensome diseases at once.

## Figures and Tables

**Table 1:** Primers and probes used in the real-time RT-PCR assays for the detection and quantification of aMPV and the detection of NDV.

Virus	Oligonucleotide	Sequence	Reference
aMPV	SH forward primer	5′-TAGTTTTGATCTTCCTTGTTGC-3′	Cecchinato et al. (2013) [18]
	SH reverse primer	5′-GTAGTTGTGCTCAGCTCTGATA-3′	
	MB SH-B probe	5′ FAM-CGCGATCATTGTGACAGCCAGCTTCACGATCGCG-3′ Iowa Black^®^ FQ	
NDV	M+4100 forward primer	5′-AGTGATGTGCTCGGACCTTC-3′	Wise et al. (2004) [19]
	M−4220 reverse primer	5′-CCTGAGGAGAGGCATTTGCTA-3′	
	M+4169 probe	5′ FAM-TTCTCTAGCAGTGGGACAGCCTGC-3′ TAMRA	

**Table 2:** The number of NDV and aMPV-positive birds based on the results of real-time RT-PCRs. Eight swabs were tested at each time point.

Days Post-Vaccination	NDV-Positive Birds					aMPV-Positive Birds			
	B1	VG/GA	TRT-B1	TRT- VG/GA	CONTROL	TRT	TRT-B1	TRT- VG/GA	CONTROL
2	8	7	8	8	0	0	1	0	0
4	8	8	7	8	0	1	0	0	0
6	8	7	8	7	0	4	1	3	0
8	7	8	7	4	0	2	0	4	0
10	7	2	4	4	0	5	2	5	0
14	0	0	0	0	0	7	7	6	0
18	0	0	0	1	0	4	8	4	0
22	0	1	0	0	0	0	4	1	0
26	0	0	0	0	0	2	1	0	0
30	0	0	0	0	0	2	2	0	0
TOTAL	38	33	34	32	0	27	26	23	0

## Data Availability

The data presented in this study are available on request from the corresponding author.
